# The Snail Family Gene *Snai3* Is Not Essential for Embryogenesis in Mice

**DOI:** 10.1371/journal.pone.0065344

**Published:** 2013-06-06

**Authors:** Cara K. Bradley, Christine R. Norton, Ying Chen, Xianghua Han, Carmen J. Booth, Jeong Kyo Yoon, Luke T. Krebs, Thomas Gridley

**Affiliations:** 1 Center for Molecular Medicine, Maine Medical Center Research Institute, Scarborough, Maine, United States of America; 2 Graduate School of Biomedical Sciences, University of Maine, Orono, Maine, United States of America; 3 Section of Comparative Medicine, Yale University School of Medicine, New Haven, Connecticut, United States of America; University of Colorado, Boulder, United States of America

## Abstract

The Snail gene family encodes zinc finger-containing transcriptional repressor proteins. Three members of the Snail gene family have been described in mammals, encoded by the *Snai1*, *Snai2*, and *Snai3* genes. The function of the *Snai1* and *Snai2* genes have been studied extensively during both vertebrate embryogenesis and tumor progression and metastasis, and play critically important roles during these processes. However, little is known about the function of the *Snai3* gene and protein. We describe here generation and analysis of *Snai3* conditional and null mutant mice. We also generated an EYFP-tagged *Snai3* null allele that accurately reflects endogenous *Snai3* gene expression, with the highest levels of expression detected in thymus and skeletal muscle. *Snai3* null mutant homozygous mice are viable and fertile, and exhibit no obvious phenotypic defects. These results demonstrate that *Snai3* gene function is not essential for embryogenesis in mice.

## Introduction

The Snail gene family encodes zinc finger proteins that function primarily as transcriptional repressors [1], [2]. Three members of the Snail gene family have been described in mammals, encoded by the *Snai1* (also called *Snail*), *Snai2* (*Slug*), and *Snai3* (*Smuc*) genes. The SNAI1 and SNAI2 proteins are key regulators of the epithelial-mesenchymal transition, directly repressing transcription of genes encoding components of cell-cell adhesive complexes in epithelia. The SNAI1 and SNAI2 proteins also have demonstrated roles in other important developmental and cellular processes, such as the protection of cells from programmed cell death, the establishment of left-right asymmetry and the regulation of cell motility [3]. *Snai2* gene expression is induced during muscle regeneration, and *Snai2* null mice exhibit defective muscle regeneration [4]. A recent study utilized ChIP-Seq and gene expression analyses to demonstrate that a Snai1-HDAC1/2 repressive complex bound and excluded the myogenic transcription factor MyoD from its targets [5]. These authors further showed that a regulatory network involving myogenic regulatory factors, Snai1/2, and the microRNAs miR-30a and miR-206 acted as a molecular switch controlling entry into myogenic differentiation.

In contrast to the *Snai1* and *Snai2* genes, much less is known about the function of the *Snai3* gene, which was cloned using a degenerate PCR-amplification protocol as a Snail family gene expressed in adult mouse skeletal muscle [6]. The human *SNAI3* gene was subsequently identified by in silico analyses [7], [8]. Originally, this gene was termed *Smuc* (for *S*nail related gene from skeletal *MU*scle *C*ells), but it has since been renamed *Snai3*. The SNAI3 protein binds to the same E2 box sequences (CAGGTG and CACCTG) bound by the SNAI1 and SNAI2 proteins, and functions as a transcriptional repressor. Northern blot analysis revealed that the *Snai3* gene was highly expressed in adult mouse skeletal muscle and thymus, was expressed at lower levels in adult heart, lung and spleen, and was also expressed during embryogenesis [6]. Analysis by in situ hybridization during mouse embryogenesis revealed that *Snai3* transcripts were first observed at embryonic day (E)13.5 in skeletal muscle and diaphragm [9]. At E15.5, in addition to skeletal muscle and diaphragm expression, *Snai3* transcripts also were expressed in the thymus. Skeletal muscle and thymus remained the dominant sites of *Snai3* expression through the early postnatal period. We describe here generation and analysis of *Snai3* null mutant mice, utilizing two different null alleles. These mice are viable and fertile, and exhibit no obvious phenotypic defects. These results demonstrate that *Snai3* gene function is not essential for embryogenesis in mice.

## Results

### Generation of Snai3^flox^, Snai3^null^, and Snai3-EYFP Mice

**Figure 1:**
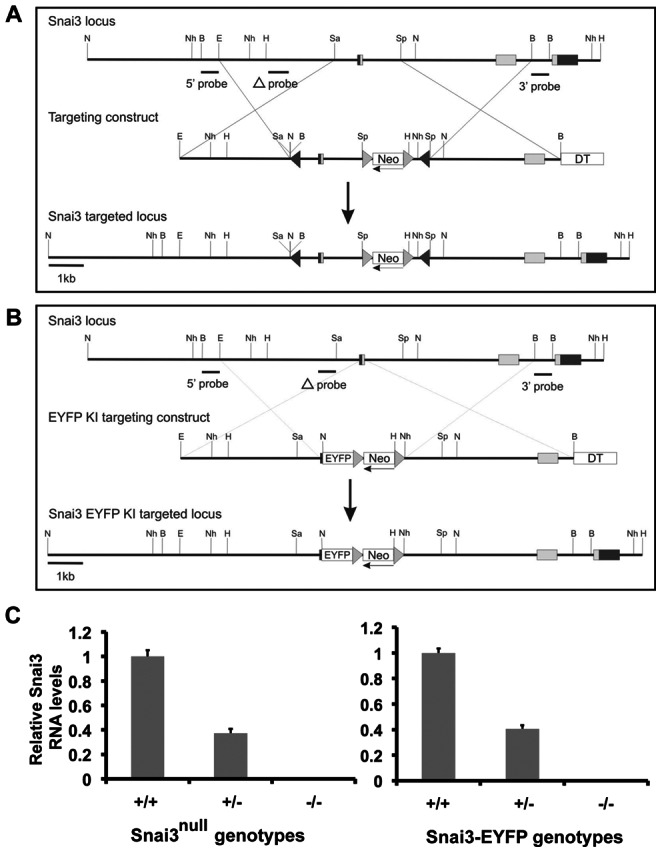
Generation of *Snai3* mutant alleles. (**A**) Construction of the *Snai3* conditional null allele. Schematic representation of the wildtype *Snai3* allele, the *Snai3^flox-neo^* targeting vector, and the targeted *Snai3^flox-neo^* allele. Exons are indicated by rectangles with coding sequences designated by gray shading, and noncoding sequences in black. LoxP sequences are marked by black triangles and FRT sites by gray triangles. An FRT-neo-FRT-loxP- cassette was inserted between exons 1 and 2, and the second loxP site was inserted 5′ to exon 1. A diphtheria toxin (DT) cassette was included for negative selection of randomly integrated clones. Hybridization probes used for Southern blot analyses are indicated. Abbreviations: B, BamHI; E, EcoRI; H, HindIII; N, NcoI; Nh, NheI; Sa, SalI; Sp, SpeI. (**B**) Construction of the *Snai3-EYFP^neo^* knock-in allele. Schematic representation of the wildtype *Snai3* allele, the *Snai3-EYFP^neo^* targeting vector, and the targeted *Snai3-EYFP^neo^* allele. Exons are indicated by rectangles with coding sequences designated by gray shading. FRT sequences are marked by gray triangles. An EYFP-FRT-neo-FRT cassette was inserted at the ATG site in exon1, replacing the remainder of the exon1 coding sequence. A diphtheria toxin (DT) cassette was included for negative selection. Hybridization probes used for Southern blot analyses are indicated. Restriction enzyme abbreviations are as in A. (**C**) Absence of *Snai3* RNA expression in *Snai3^null^/Snai3^null^* and *Snai3-EYFP*/*Snai3-EYFP* embryos. Quantitative RT-PCR of relative *Snai3* transcript levels in RNA isolated from *Snai3^null^/Snai3^null^*, *Snai3-EYFP*/*Snai3-EYFP* and littermate control embryos revealed the absence of *Snai3* transcripts in the homozygotes, confirming that both alleles were *Snai3* null alleles.

In order to assess whether the *Snai3* gene plays an essential in vivo role in mice, we created three *Snai3* targeted mutant alleles: a *Snai3^flox^* allele for conditional *Snai3* gene inactivation (Fig. 1A), a *Snai3^null^* allele, and a *Snai3-EYFP* knock-in allele that is also a *Snai3* null allele (Fig. 1B). The *Snai3^null^* allele was generated by *Cre* recombinase-mediated deletion of the *Snai3^flox^* allele, which results in deletion of *Snai3* promoter sequences and the first exon of the *Snai3* gene. In the *Snai3-EYFP* allele, EYFP coding sequences replace *Snai3* coding sequences contained in the first exon of the *Snai3* gene. Mice homozygous for all three *Snai3* alleles were viable and fertile. To confirm that the *Snai3^null^* and *Snai3-EYFP* alleles were truly null alleles, we harvested RNA from *Snai3^null^*/*Snai3^null^* and *Snai3-EYFP*/*Snai3-EYFP* homozygous mutant embryos and littermate controls at E14–E15 (Fig. 1C). No *Snai3* transcripts were detected in either *Snai3^null^*/*Snai3^null^* or *Snai3-EYFP*/*Snai3-EYFP* homozygotes, confirming that both alleles are *Snai3* null alleles.

### The *Snai3-EYFP* Allele Accurately Reflects Endogenous *Snai3* Gene Expression

**Figure 2:**
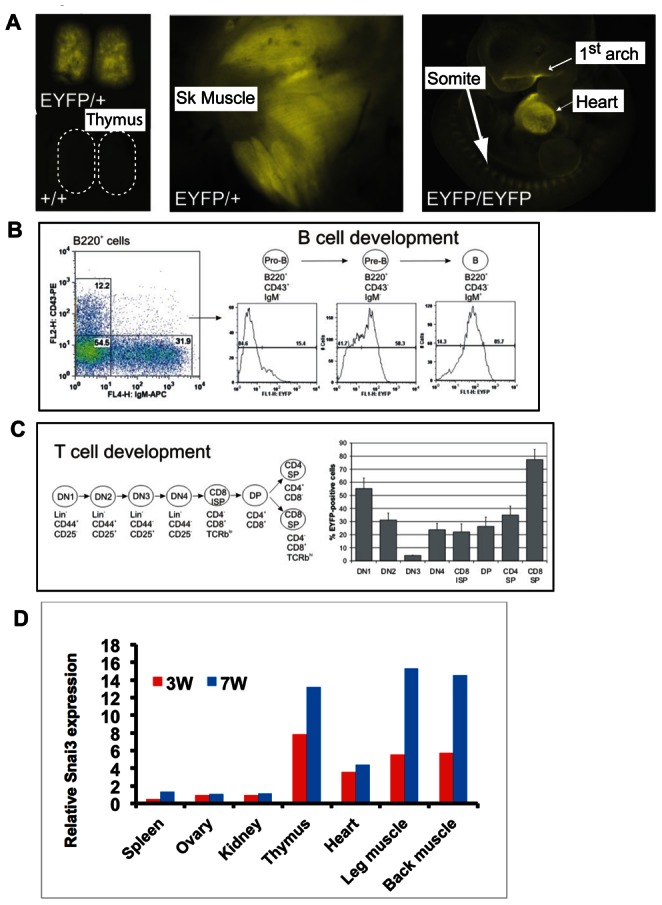
Expression of the *Snai3-EYFP* allele. (**A**) EYFP expression is observed in thymus and in leg skeletal muscle at E15.5, and in heart, first branchial arch and somites at E10.5. EYFP expression was visualized by fluorescent stereomicroscopy. Dotted lines in the left panel outline the wild type thymus lobes. (**B**) Flow cytometry analysis demonstrated EYFP expression in Pre-B and B cells, but not in Pro-B cells of adult mouse bone marrow. (**C**) Flow cytometry analysis of thymus demonstrated EYFP expression in most T cell subsets. EYFP expression was highest in CD8 single positive T cells. (**D**) Quantitative RT-PCR of endogenous *Snai3* RNA expression in the indicated organs of three (red bars) and seven (blue bars) week old wild type mice. Expression was normalized to βeta actin RNA levels in each organ.

We assessed expression of the *Snai3-EYFP* allele to determine how closely it matched *Snai3* RNA expression. The sites of highest *Snai3* RNA expression are skeletal muscle and thymus [6], [9]. By fluorescent stereomicroscopy, *Snai3-EYFP* expression was readily detected in both embryonic thymus and skeletal muscle at E15.5 (Fig. 2A). At E10.5, expression also was detected in both heart and the maxillary and mandibular portions of the first branchial arch (Fig. 2A). Both these sites of expression have been reported previously [6], [10]. We used flow cytometry to examine in bone marrow cells and thymocytes expression of both the *Snai3-EYFP* allele and lineage markers of lymphoid cells. As expected [8], [9], *Snai3-EYFP* expression was evident in both T and B cell populations (Fig. 2B, C). In the bone marrow, greater than 90% of EYFP-positive cells also expressed the pan B cell specific marker B220 (Fig. 2B). Notably, the percentage of EYFP-positive cells was greater in more mature B cell populations. Within the thymus, EYFP-positive cells were evident in all subpopulations during T cell development (Fig. 2C). However, the percentage of EYFP-positive cells was greater in very early T cells (DN1), as well as mature CD8 single positive T cells (80%). All sites of *Snai3-EYFP* expression were also sites of *Snai3* RNA expression, as detected by quantitative RT-PCR of organs from adult mice (Fig. 2D), demonstrating that the *Snai3-EYFP* allele is a useful and accurate reporter of *Snai3* gene expression.

### *Snai3* Mutant Mice do not Exhibit an Obvious Phenotype

**Figure 3:**
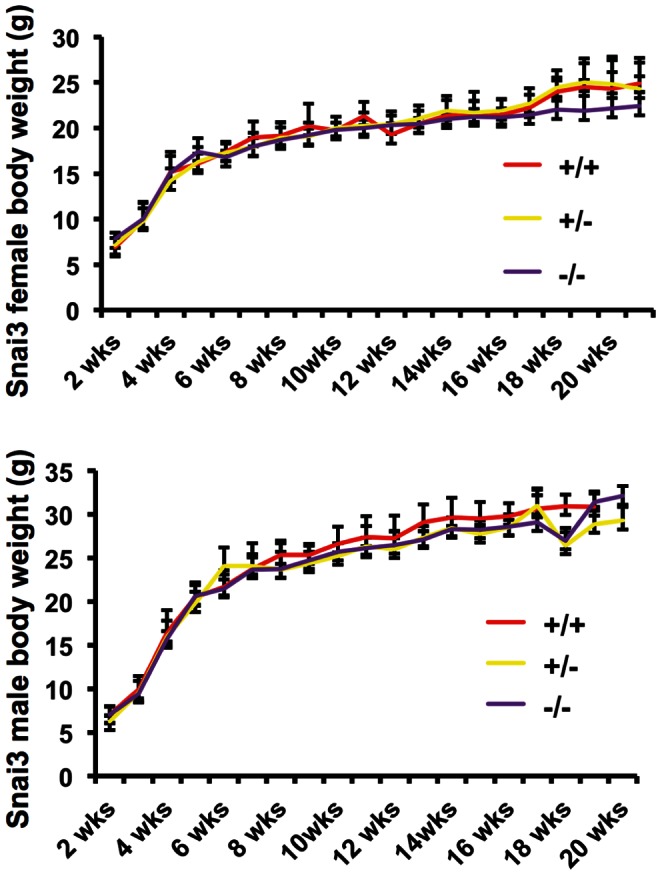
*Snai3^null^/Snai3^null^* homozygous mice exhibit normal postnatal growth. Growth curves of *Snai3^null^/Snai3^null^* homozygous, *Snai3^null^/+* heterozygous and wild type littermate mice. Weights (in grams) were plotted against age (in weeks). Data presented are from at least four mice in each group. Error bars indicate the standard deviations.

**Figure 4:**
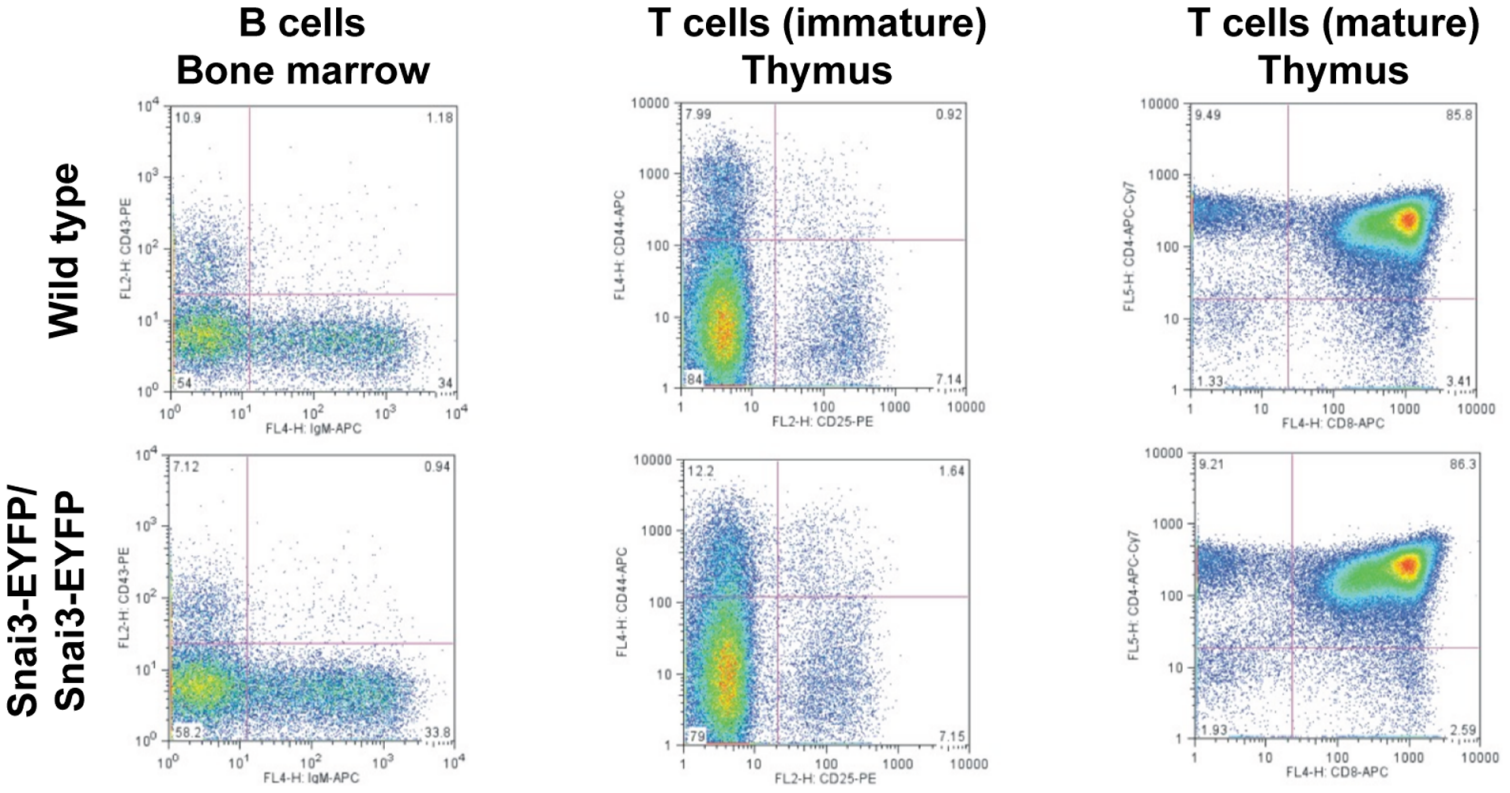
*Snai3-EYFP/Snai3-EYFP* homozygous mice do not exhibit altered lymphoid differentiation. Flow cytometry analyses of cells from bone marrow and thymus demonstrated that *Snai3-EYFP/Snai3-EYFP* homozygous mice (*n* = 3) did not exhibit obvious defects in differentiation of B, immature T, or mature T cells.

*Snai3^null^* and *Snai3-EYFP* homozygous embryos and mice did not exhibit any obvious phenotype. Both *Snai3^null^*/*Snai3^null^* (Table 1) and *Snai3-EYFP*/*Snai3-EYFP* homozygotes were born at expected Mendelian frequencies. Analysis of postnatal growth curves showed no differences in the rate of growth of either male or female *Snai3^null^* homozygotes versus their heterozygous and wild type littermate controls (Fig. 3). Flow cytometry analyses of bone marrow cells and thymocytes from *Snai3-EYFP* homozygous mice did not reveal obvious defects in differentiation of B and T cells (Fig. 4). Since neural crest cell-specific deletion of the *Snai1* gene on a *Snai2* null genetic background results in craniofacial defects [11], and the *Snai3* gene is expressed in the first branchial arch (Fig. 2A), we examined alcian-blue/alizarin-red stained skeletons from *Snai3^null^* homozygous and littermate control mice. Analysis of these skeletal preparations did not reveal any obvious defects in the cranial, axial or limb skeletons of *Snai3^null^*/*Snai3^null^* mice (data not shown). We also performed extensive histopathological and clinical chemistry analyses of *Snai3^null^* homozygous and wild type littermate control mice at eight to ten months of age. These analyses did not reveal any abnormal phenotype reproducibly present in the *Snai3^null^*/*Snai3^null^* mice. We conclude that the *Snai3* gene is not essential for embryogenesis and normal development in mice.

**Table 1:** *Snai3^null^*/*Snai3^null^* mice are born and survive at the expected Mendelian frequency.

			Genotype	
Number of litters	Number of mice	+/+	*Snai3^null^*/+	*Snai3^null^*/*Snai3^null^*
30	201	52 (25.9%)	93 (46.3%)	56 (27.8%)

*Snai3^null^*/+ mice were intercrossed, and progeny genotypes were determined at weaning.

## Discussion

Relatively little is known about the function of the *Snai3* gene and protein, compared to the numerous studies of its family members *Snai1* and *Snai2*. The *Snai3* gene was originally isolated as a Snail family gene expressed in adult mouse skeletal muscle [6]. The SNAI3 protein bound the same E2 box sequence (CAGGTG and CACCTG) recognized by the SNAI1 and SNAI2 proteins, and functioned as a transcriptional repressor. Northern blot [6] and in situ hybridization [9] analyses indicated that the *Snai3* gene was expressed at highest levels in thymus and skeletal muscle. We confirmed these and other sites of expression by analyzing expression of the *Snai3-EYFP* allele (Fig. 2).

We demonstrated by qRT-PCR that the *Snai3^null^* and *Snai3-EYFP* alleles generated no stable *Snai3* transcripts, indicating that both are *Snai3* null alleles (Fig. 1C). We were surprised to find that neither the *Snai3^null^*/*Snai3^null^* nor the *Snai3-EYFP*/*Snai3-EYFP* homozygotes exhibited an obvious mutant phenotype. It should be noted that the absence of an obvious phenotype in *Snai3* homozygous mutant mice in the laboratory environment does not preclude an essential role for the *Snai3* gene in mice inhabiting their natural environment. However, subsequent to our initiation of these studies, a potentially relevant finding was made from a comparison of the requirements for Snail family genes in fish. While *Snai3* genes are present in both the zebrafish and pufferfish (fugu) genomes, the medaka genome does not contain a *Snai3* gene [12], indicating that *Snai3* gene function also is not required for embryonic development in teleost fish (at least in medaka).

We have previously demonstrated genetic redundancy of the *Snai1* and *Snai2* genes in mice utilizing two different Cre driver lines for conditional *Snai1* gene deletion on either wild type or *Snai2* null genetic backgrounds, *Wnt1-Cre* (for neural crest cell deletion) [11] and *Prrx1-Cre* (for deletion in limb bud mesenchymal stem cells that give rise to chondrogenic precursors of the limb long bones) [13]. In both cases, cartilaginous precursors of endochondral bones were shorter in the *Snai1/Snai2* double mutant mice, although neither of these genes was essential for delamination of neural crest cells from the dorsal neural tube through E9.5 [14]. During long bone development, our work clearly demonstrated that the *Snai1* and *Snai2* genes could compensate quantitatively, temporally and spatially for the other genes’ loss [13]. A similar mechanism may explain the absence of an obvious mutant phenotype in the *Snai3* null mice. However, testing the functional equivalence of the SNAI1, SNAI2 and SNAI3 proteins in mice ultimately will require the generation and analysis of a series of knock-in alleles, placing the coding sequence for each Snail family member under the transcriptional control of a different family member.

A recent gain-of-function study in mice demonstrated that expression of *Snai3* by retroviral transduction of hematopoietic stem cells in bone marrow chimeras resulted in a block in lymphoid cell development [15]. However, we did not detect any obvious defects in lymphoid cell differentiation in *Snai3* null mice. Two alternatives could explain the absence of a phenotype in lymphoid cells in the *Snai3* null mutants. Gain-of-function studies yield phenotypes in cells that are competent to respond to the protein or signal being misregulated, but do not in and of themselves prove that this gene is required for their development in the wild type situation. Alternatively, there could be compensatory regulation (of the sort observed in long bone chondrogenesis) by another Snail family gene member, or even by an unrelated gene(s).

## Materials and Methods

### Ethics Statement

All experimental procedures performed on mice were in accordance with the recommendations of the Guide for the Care and Use of Laboratory Animals of the National Institutes of Health, and were approved by the Institutional Animal Care and Use Committee at Maine Medical Center (Protocol number 1023).

### Construction of the *Snai3^flox-neo^* Allele

To generate the *Snai3^flox-neo^* targeting construct, an 8 kb EcoRI/BamHI fragment (subcloned from 129S7/AB2.2 BAC clone bMQ134F24; Wellcome Trust Sanger Institute) containing a portion of the *Snai3* genomic locus was cloned into pBluescript II SK with a modified multiple cloning site (SacI-EcoRI-BamHI-MluI-NotI-XhoI). The 5′ distal loxP site was generated by insertion of a synthetic oligonucleotide cassette at the SalI site. A FRT-neo-FRT-loxP cassette was inserted at the SpeI site, and a diphtheria toxin-A cassette was inserted at the MluI/NotI sites to permit negative selection against random integration of the targeting vector. *Cre* recombinase-mediated deletion will result in deletion of *Snai3* promoter sequences and the first exon of the *Snai3* gene.

### Construction of the *Snai3-EYFP^neo^* Allele

To generate the *Snai3-EYFP^neo^* targeting construct, the same 8 kb EcoRI/BamHI fragment described above was cloned into pBluescript II SK with a modified multiple cloning site (SacI-EcoRI-BamHI-MluI-NotI-XhoI). A diphtheria toxin-A cassette was inserted at the BamHI/MluI sites. Using bacterial recombineering, the coding region of exon 1 (ATG onwards) of the *Snai3* gene was replaced with an “EYFP-loxP-neo-loxP” cassette. This EYFP-loxP-neo-loxP cassette was generated by PCR amplification of a vector in which the EYFP gene from pEYFP-N1 (Clontech) was inserted upstream of the loxP-neo-loxP at the SacII/XhoI sites.

### Electroporation of ES Cells and Generation of Mutant Mice

CJ7 ES cells [16] were electroporated with linearized *Snai3^flox-neo^* or *Snai3-EYFP^neo^* targeting vector, placed under selection in G418, and screened for homologous recombination by Southern blot hybridization. Correctly-targeted ES cell clones were injected into C57BL/6J blastocysts to generate chimeric mice. Male chimeras were mated to female mice to obtain germline transmission of the *Snai3^flox-neo^* and *Snai3-EYFP^neo^* alleles. *Snai3^flox-neo^/+* heterozygous mice were mated to the *Meox2-Cre* deleter line [17] to excise *Snai3* genomic sequences between the loxP sites and generate the *Snai3^null^* allele. *Snai3-EYFP^neo^*/+ heterozygous mice also were mated to the *Meox2-Cre* deleter line to excise the loxP-neo-loxP cassette and generate the *Snai3-EYFP* allele. *Snai3^flox-neo^/+* heterozygous mice were mated to a deleter line expressing the Flpe recombinase [18] to excise the FRT-neo-FRT cassette and generate the *Snai3^flox^* allele.

### Mouse and Embryo Genotyping

For genotyping *Snai3-EYFP* mice and embryos, PCR primers for the *Snai3-EYFP* mutant allele are Snai3-F2 (CTGGTTGGCTGAGGTGGTGCGCTAT) and EYFP-R2 (CTTGCCGGTGGTGCAGATGAA), with a product size of 306 base pairs. Genotyping primers for the wild type *Snai3* allele (for use in conjunction with the *Snai3-EYFP* mutant allele primer set above) are Snai3-F2 and Snai3-R3 (TGTTGAAATGGAAAACTCTAGCCCCTTC), with a product size of 354 base pairs.

For genotyping *Snai3^flox^* mice and embryos, PCR primers for the *Snai3^flox^* allele are Snai3-loxP-F (GCAGCCAGCAGGAATGTGTCCTCAGAT) and Snai3-loxP-R (AGTCGGCAGCGTAGGAGACAGT), with a product size of 326 base pairs. This same primer set will amplify a product of 266 base pairs from the wild type *Snai3* allele (this primer set flanks the region including the 5′ loxP site in the *Snai3^flox^* allele).

Primers for the *Snai3^null^* allele (i.e., the deleted form of the *Snai3^flox^* allele) are Snai3-loxP-F and Snai3-delR (AAGCTGGTATGTGCTCTCCAAGTGC), with a product size of 316 base pairs.

All PCR reactions were performed using ThermoPrime ReddyMix PCR Master Mix (Fisher Scientific) and the following cycling conditions: 94°C, 3 minutes; [94°C, 30 seconds; 60°C, 45 seconds; 72°C, 45 seconds]×40 cycles; 72°C, 4 minutes; 4°C hold.

### Analysis of Snai3-EYFP Expression

EYFP fluorescent protein expression was analyzed in whole mount embryos through E10.5, and in partially dissected embryos at later stages. No differences were noted in expression of the *Snai3-EYFP^neo^* or *Snai3-EYFP* alleles. Embryos were analyzed and digital photographs taken on a Zeiss Discovery V12 fluorescence stereomicroscope. Flow cytometry for EYFP and marker protein expression was performed on suspensions of bone marrow and thymus from adult mice, as described previously [19].

### Analyses of *Snai3* Mutant Mice

Alcian blue-alizarin red-stained skeletal preparations were generated as described previously [11]. Blood for clinical chemistry analyses, and tissues from all major organ systems of male *Snai3^null^*/*Snai3^null^* homozygous (*n* = 5) and wild type littermate control (*n* = 3) mice, as well as the reproductive tracts of an additional four homozygous and three wild type female mice, were harvested and processed for histopathological analysis. Soft tissues were fixed in neutral buffered 10% formalin (Fisher Scientific). Limbs and the skull with brain were fixed in Bouin’s solution (Ricca Chemical Company). Tissues were processed, embedded in paraffin, and sectioned by routine methods (Yale Mouse Research Pathology, Section of Comparative Medicine, Yale University School of Medicine). Tissues were sectioned at 5 microns and stained with hematoxylin and eosin. Tissues were examined by routine light microscopy with an Axio Imager A1 microscope (Carl Zeiss Micro Imaging). Necropsy, histopathology, and clinical chemistry examination were performed blind to experimental genotype. Clinical chemistry assays were performed using standard methods by Antech Diagnostics (Irvine, CA). Flow cytometry to assess lymphoid differentiation was performed as described previously [19].

### Quantitative RT-PCR

Organs from three- or seven-week old wild type mice, and embryos at E14–E15, were dissected and immersed in RNAlater (Ambion). Genotypes were identified from DNA samples by allele-specific PCR. Total RNA was isolated using the Qiagen Mini mRNA Extraction kit. RNA (2 µg of each sample) was reverse-transcribed with random hexamer primers (Ambion). Six nanograms of cDNA were used for real-time PCR amplification for each well, using primer sequences from Primerbank. qRT-PCR was performed using Super SYBR Green PCR Master Mix on a 7500 Real Time PCR system (Applied Biosystems) using SDS software. For each gene tested we performed three experimental replicates and four biological replicates. *Snai3* primer sequences were CTGCCCTGCATCTGTAAGGT (forward primer, located in exon 2) and TGGTACCAACGTGAGTCTGC (reverse primer, located in exon 3). For comparison of *Snai3* expression in various organs of three- or seven-week old wild type mice, gene expression levels were normalized to the βeta actin RNA level. For comparison of *Snai3* expression in wild type, heterozygous and homozygous *Snai3* mutant embryos, the βeta actin-normalized *Snai3* RNA level in the wild type embryos was set to 1.0, to which the βeta actin-normalized *Snai3* RNA levels of the heterozygous and homozygous *Snai3* mutant embryos were compared.
